# Analysis of resources assisting in coping with swallowing difficulties for patients with Parkinson’s disease: a cross-sectional study

**DOI:** 10.1186/s12913-016-1467-6

**Published:** 2016-07-18

**Authors:** Aiko Matsushima, Junichi Matsushima, Akihisa Matsumoto, Fumio Moriwaka, Sanae Honma, Kazunori Itoh, Keiko Yamada, Shun Shimohama, Hirofumi Ohnishi, Mitsuru Mori

**Affiliations:** Department of Public Health, Sapporo Medical University, School of Medicine, Nishi 17-chome, Minami 1-jo, Chuo-ku Sapporo, 060-8556 Japan; Department of Neurology, Jyouzankei Hospital, Sapporo, Japan; Hokuyukai Neurological Hospital, Sapporo, Japan; Iwamizawa Neurological Medical Clinic, Iwamizawa, Japan; Department of Neurology, Sapporo Medical University, School of Medicine, Sapporo, Japan; ENT Clinic, Sapporo, Japan

**Keywords:** PD, Swallowing difficulty, Dietary modification, Care foods, Costs, Caregivers, BMI

## Abstract

**Background:**

Malnutrition induced by swallowing difficulties (SD) impairs the quality of life and gives rise to SD-related costs in Parkinson’s disease (PD) patients. With results of a swallowing difficulty questionnaire and data of resources specifically obtained such as SD-related costs, caregivers, and dietary therapies, this study is to suggest statistically supported ideas for improvements in arrangements for how participants cope with SD and maintain general well-being.

**Methods:**

We interviewed 237 PD patients. The SD-related costs involved those incurred by the provision of dietary modifications, care oriented foods, alternatives, and supplements. Dietary therapies included rice porridge and commercially available care foods. The relationships between BMI (body mass index) and the severity of SD assumed in this paper as indicators for general well-being and as resources for coping with SD for PD patients were statistically analyzed.

**Results:**

A lower BMI was found in participants eating porridge consistency rice (*p* = 0.003) and eating porridge rice is significantly related to the severity of SD (*p* < 0.0001) and PD (*p* = 0.002). The severity of SD increased with age and PD duration (*p* = 0.035, *p* = 0.0005). Outlays for dietary modifications are the lowest reported here (*p* < 0.004) but the number of participants using dietary modifications is the largest among the SD-related items (*n* = 58). Eating care foods were reported for 11 older participants (*p* < 0.0001), most female (10/11). No lower BMI was found in participants eating care foods when compared with participants eating ordinary foods. Dietary modifications were performed by caregivers (OR: 6.8, CI: 3.1-15.2, *p* < 0.0001) and were related to the presence of children (OR: 3.4, CI: 1.2-11.4. *p* = 0.024). Older participants commonly live with spouses and children.

**Conclusions:**

Severe SD is associated with higher costs of coping with SD. A lower BMI is associated with modified foods, mostly eaten to cope with SD. Presence of caregivers and other persons residing with the participants here are related to dietary modifications but not to care food-related costs. Care foods may be effective in preventing malnutrition although the number who are able to cover the added expenses is limited because of the higher prices and shortage of information on the usefulness of care foods.

**Electronic supplementary material:**

The online version of this article (doi:10.1186/s12913-016-1467-6) contains supplementary material, which is available to authorized users.

## Background

Parkinson’s disease (PD) is a complex progressive neurogenic disease with motor and a wide variety of non-motor symptoms which strongly affect the quality of life and health-related outlays [1]. Swallowing difficulties are considered to be an important complication in PD. A meta-analysis has shown that objectively determined, the prevalence is up to 80 % [2], however, data based on subjective assessments vary, with a pooled prevalence estimate of 35 % from a random effect analysis. The wide variations in the prevalence of dysphagia using subjective assessments was explained by differences in gender, age, disease duration and dementia [3]. This discrepancy in the prevalence of dysphagia showed that dysphagia is common in PD, patients do not always report swallowing disturbances unless asked [2]. Even if asked, the majority of participants reporting “no difficulty in swallowing” showed significant changes in swallowing function using a simple bedside water test [4].

Nutritional problems caused by swallowing difficulties and aspiration are a cause of aspiration pneumonia and may be lethal [5] so adequate support for dysphagia is crucial for maintaining quality of life (QOL) and survival. Once dysphagia occurs in PD, long-term support to assist with swallowing is needed [6, 7]. Malnourished PD patients have been reported to have a poorer QOL than well-nourished patients [8] and improvements in nutritional status resulted in quality of life improvements [9]. Attention to nutritional status is an important component in the quality of life and therefore in the total care of PD patients [10]. The etiology of malnutrition is multi-factorial and in addition to depression [6] may come from loss of appetite, constipation, early satiety, and swallowing problems as reported on the scored PG-SGA (Patient Generated Subjective Global Assessment) [11]. For the effects of body weight loss on health-related QOL, a prospectively compiled longitudinal PD outcomes data study showed that weight appears to correlate with health-related QOL. An objective assessment showed that self-reports like “no difficulty in swallowing” is not at all a reliable indicator of swallowing ability [4]. An early prediction of weight loss and poorer QOL, therefore, would help caregivers and practitioners improve patient management and expectations [12].

To cope with SD in PD patients provisions related to costs, types of foods eaten by patients, and caregivers all play important roles. To cope with SD, costs are incurred, however reports on costs of daily necessities incurred in relation to swallowing were not considered in previous socio-economic reports [13]. There are a few micro-costing studies on oral nutritional supplements at the hospital level, but in these the main indicator of healthcare resource use was mortality, complications, and length of hospital stay [14]. For the costs of malnutrition at the national level, calculations represent public expenditures arising with health maintenance and social care of patients with diet-related malnutrition. For the calculation of public expenditures, data for the prevalence of disease-related malnutrition (DRM) vary in hospital out-patient and primary-care clinics [15]. The estimated DRM represent the public expenditures only but do not consider out of pocket outlays due to DRM.

For costs of coping with SD, other than medical expenses, at the individual level, there would be two categories. One, those incurred as a result of SD and the other aimed at improving well-being. The former includes dietary modifications and commercially available care oriented foods (care foods) and the latter relates to food supplements and alternative therapies like massage or acupuncture. The SD-related to PD is considered to arise due to impairment in the sequence of swallowing in the oral and pharyngeal phase in PD patients. Normal swallowing is a complex mechanism involving voluntary and reflex events that is governed by an afferent system, the brain stem swallowing center and the efferent motoneural system [16]. The pathophysiology of dysphagia in Parkinson’s disease is complex and dependent on coordination among both peripheral and central nervous system factors. Therefore, for SD sufferers, modified diets should be considered as a means of alleviation which participants and/or caregivers can manage [17]. Where it is not possible to manage malnutrition using dietary modifications, care foods would be an alternative choice [18]. As PD is a progressive neuro-degenerative disease where PD related disabilities require personnel to assist in the activities of daily living and sometimes needs caregiver support at meals to effect the dietary modifications and/or serve the food to patients in advanced stages of PD.

The first objective in the current study is to identify factors related to SD using statistical analysis. The second objective is to determine the relationships between the factors. Then based on the results the third objective is to understand the behavior patterns behind the food types selected to cope with malnutrition, as indicated by lower BMI and SD values. Finally the study will consider the severity of SD and PD in participants and describe optimal characteristics of caregivers for patients with advanced PD and SD.

## Methods

The interview period was from February to October in 2013. Among 249 PD (Parkinson’s disease) patients visiting two hospitals (Department of Neurology, Jyouzankei Hospital and Hokuyukai Neurological Hospital) and one clinic (Iwamizawa Neurological Medical Clinic), 245 patients participated in this study (Additional files 1 and 2). Participants living at care facilities and/or under public livelihood assistance were excluded and 237 PD patients (82 males and 155 females) were interviewed for data collection by members of the nursing staff. All participants signed an informed consent form before answering the questionnaire. The ages ranged from 45.1 to 91.3 (mean: 71.3, Standard Deviation: 8.3) and the duration of suffering from PD was from 0.3 to 36.2 years (mean: 8.32, Standard Deviation: 6.5). Swallowing difficulties were evaluated with the Swallowing Difficulty Questionnaire (SDQ) [19]. Our questionnaire in this study was focused on SD-related costs such as eating utensils, commercially available care oriented foods (care foods) and dietary modifications, as well as the costs incurred in maintaining well-being and promoting healthcare such as food supplements and alternatives. Here care foods included two kinds of commercially available care foods in Japan. One is a home delivery lunch box, in which provisions of foods are processed based on the degree of impairment of the dysphagia. The other is a la carte foods such as softened fish, bits of vegetables, meat and similar which are packed in a plastic bag. Cooked dishes in a plastic bag are also served for use by recipients. Participants here were not asked which care foods, a home delivery lunch box or a la carte foods, are involved.

Participants with scores based on the SDQ at and above 11.0 points were diagnosed as showing the presence of swallowing difficulty (SD). The severity of the PD was determined with the Hoehn & Yahr (H&Y) rating scale [20] and participants at stage 3 and higher were defined as suffering from advanced PD. For the statistical analysis these scales were assigned numbers from 1 to 7 based on the modified H&Y rating scale [20] (Additional file 3).

The SD-related costs were divided into 5 categories based on the monthly outlays involved, below 5 thousand yen (about 40–50 US dollars at 2015 exchange rates), 5 to 10 thousand yen, 10 to 15 thousand yen, 15 to 20 thousand yen, and above 20 thousand yen. For the statistical analysis these categories were assigned values from 1 to 5. Information of annual incomes were also obtained, and divided into 4 categories, below 2 million yen (sixteen to twenty thousand US dollars), 2 to 4 million yen, 4 to 6 million yen, and above 6 million yen. These categories were assigned values from 1 to 4 for the statistical analysis. The dietary modifications were assigned values as follows, participants eating ordinary cooked rice (1 point), ordinary rice with processed side dishes (2 points), and rice porridge (3 points).

### Statistics

The nonparametric Spearman’s rank correlation coefficient was used to assess the relationships of the various dependent variables. Comparison of the mean values was performed by Welch’s *t*-test and Wilcoxon signed rank coefficient. Statistics for 2-by-2 tables used Fisher’s exact test or the chi 2 test. A logistic regression analysis and the least squares method are used for goodness of fit for some variables. The software used for the statistics was the JMP 10.0.2 developed by SAS Institute Inc. This study was approved by the ethics committees of Sapporo Medical University (No. 24-2-73) and the Department of Neurology, Jyouzankei Hospital, as well as the Hokuyukai Neurological Hospital and Iwamizawa Neurological Medical Clinic.

## Results

Table 1 shows that there are no statistically significant differences in gender and BMI between the non-SD (under 11.0 points) and SD (at and above 11.0 points) participants based on the SDQ [19]. There are no significant differences in outlays for SD-related costs other than eating utensils between the non-SD and SD groups. The PD duration and age were significantly higher in SD than in the non-SD participants. For the score of H&Y, the percentage of participants at and above stage 3 is significantly higher in the SD than in the non-SD groups. The number of participants reporting to be working is higher among participants without SD than with SD. Nobody reporting work participation used commercially available care foods. The incidence of caregivers is higher among participants with SD than among participants without SD. The score for dietary modifications is positively related to age, outlays for supplements, and presence of caregivers, but negatively related to the BMI.

**Table 1:** Basic data from the SDQ by Cohen & Manor and the scores for dietary modifications

		non SD (Under 11.0 points)		SD (At & over 11.0 points)			Score of dietary modifications goodness of fit
Items		*n*	%	*n*	%	*p* value	*t* value
Gender	Male	56	32.2	26	41.3	0.218	
	Female	118	67.8	37	58.3		ns
	Total	174	100	63	100	237	
Age	mean ± S.D.	174	70.7 ± 8.4	63	73.2 ± 7.7	0.035*	4.49 b
Duration of sickness (years)	mean ± S.D.	174	7.3 ± 6.0	63	11.0 ± 7.3	0.0005*	ns
Severity (H&Y scale)	At & above 3	84	48.3	48	76.2	0.0001*	
	Under 3	90	51.7	15	23.8		ns
	Total	174	100	63	100	237	
Work status	No	151	86.7	61	96.8	0.030*	
	Yes	23	13.3	2	3.2		ns
	Total	174	100	63	100	237	
BMI	mean ± S.D.	174	22.5 ± 3.5	63	22.0 ± 3.8	0.452	−3.41 b
Outlays of							
Eating utensils	mean ± S.D.	174	0.17 ± 0.46	63	0.54 ± 1.13	0.014*	ns
Supplements	mean ± S.D.	174	0.36 ± 0.92	63	0.66 ± 1.21	0.081	2.63 a
Presence of caregivers (*n* = 36)	No	157	90.2	44	69.8	0.0003*	4.39 b
	Yes	17	9.8	19	30.2		
	Total	174	100	63	100		

a: *p* < 0.01, b: *p* < 0.001. *: significant, ns: non-significant, S.D.: Standard Deviation

For SD-related items, median expenses and the overall mean ± Standard Deviation are as shown in Table 2. The median annual income is 2 to 4 million yen, the second lowest income category in the questionnaire. The mean income (2.3 ± 0.9) reported by participants still working (*n* = 24) is significantly higher than that (1.8 ± 0.7) for non-working participants (*n* = 210; *p* = 0.0065), these data are not shown in Table 2. For the overall mean expenses, that for care foods is the highest among the SD-related expenses. The median outlays for care foods were 5 to 10 thousand yen monthly, higher than for other SD-related items. The median outlays for all SD-related costs other than the costs of care foods are below 5 thousand yen (about 40–50 US dollars), the category with the lowest outlays in the questionnaire. The mean outlays for dietary modifications is significantly lower than the outlays for eating utensils (*p* = 0.0039), supplements (*p* < 0.0001), alternatives (*p* < 0.0001), and care foods (*p* < 0.0001). Outlays for care foods are higher than the SD-related outlays such as for eating utensils and dietary modifications, *p* = 0.0147, *p* < 0.0001. However, only few participants report eating care foods (*n* = 11), a much lower number than the number of participants reporting expenses for the other categories (n=58) as shown in Table 2. Ages of participants eating care foods and reporting outlays for dietary modifications are significantly higher than those not reporting these items. For participants with SD-related items, the presence of caregivers is statistically significantly associated with outlays for eating utensils by the logistic regression analysis. In Table 2 the presence of child (ren) (*n* = 80) included the presence of one child only (*n* = 32) and a child and spouse pair (*n* = 48). For participants residing with a child there is a negative relation with outlays for supplements. Participants residing with a spouse only are the only group that is significantly associated with incomes.

**Table 2:** Characteristics and goodness of fit for SD-related outlays and incomes

	Categories		Values		Age			Goodness of fit for presence of		
Items	median (Yen)	*n*	mean ± S.D.	*n*	mean ± S.D.			Caregivers *n* = 36	Spouse *n* = 112	Child *n* = 80
			Overall		+	-	*P*	OR, CI95, *p*	OR, CI95, *p*	OR, CI95, *p*
Incomes	2 to 4 million	115	1.85 ± 0.7	234	60.0 ± 9.2 working	72.6 ± 7.3 non-working	<0.0001*	ns	1.80, 1.25-52.6, <0.0001*:	ns
Eating utensils	<5,000	31	1.50 ± 1.0	42	72.1 ± 7.7	71.2 ± 8.5	0.4731	3.7, 2.1-7.3, <0.0001*	ns	ns
Care foods	5 to 10 thousand	3	2.55 ± 1.7	11	77.8 ± 3.7	70.6 ± 8.4	<0.0001*	ns	ns	ns
Dietary modifications	<5,000	55	1.16 ± 0.7	58	74.2 ± 7.4	69.8 ± 8.4	0.0002*	ns	ns	ns
Alternatives	<5,000	35	1.64 ± 1.0	59	70.8 ± 7.1	71.5 ± 8.7	0.5050	ns	ns	ns
Supplements	<5,000	28	1.96 ± 1.3	53	71.0 ± 9.1	71.4 ± 8.1	0.7687	ns	ns	0.71, 0.48-1.41, 0.031*

Abbreviation in table: + : for participants reporting this item; - : participants not reporting this; <: at or below the figure indicated

S.D.: Standard Deviation, *: significant

The relationships among SD-related costs and scores of SD and dietary modifications in all of the 237 participants were calculated using Spearman’s coefficient correlation analysis and the results are shown in Table 3. The negative relationship of BMI explained as malnutrition is related to the score for dietary modifications and not to the score for SD or to other SD-related items. The SD score is related to all of the SD-related outlays other than the outlays for dietary modifications, overall resulting in the higher SD-related outlays with SD. The SD-related items can be divided into two factors based on how items are related to SD. One for the management of SD like the outlays for eating utensils, care foods, and dietary modifications that are strongly related to SD, and the other for promoting health care and well-being like the expenses for alternatives and supplements although those items are related to the SD score. The outlays for eating utensils are related to outlays for care foods. The outlays for dietary modifications are not related to any other costs or to the SD score. The outlays for supplements are related to the score of SD and outlays for alternatives. Incomes are not related to any of the SD-related costs.

**Table 3:** Relations among SD-related outlays and scores, with incomes and BMI

Incomes & Outlays of	(1)	(2)	(3)	(4)	(5)	(6)	(7)	(8)	(9)
Incomes (1)	1	0.0382	0.0028	−0.0025	−0.0059	0.0030	0.0978	−0.0525	0.0570
Eating utensils (2)		1	0.2155***	0.0328	0.0855	0.2096**	0.1490*	0.2355***	−0.1036
Care foods (3)			1	−0.0250	0.0445	0.0825	0.1349*	0.2453***	0.0053
Dietary modifications (4)				1	0.0938	0.0168	0.0682	−0.0232	0.0682
Supplements (5)					1	0.2108**	0.1488*	0.0678	0.1014
Alternatives (6)						1	0.1342*	0.0355	0.0160
Score of SD (7)							1	0.3913***	−0.0548
Score of modifications (8)								1	−0.2184***
BMI (9)									1

*: *p* < 0.05; **: *p* < 0.001; ***: *p* < 0.0001, the numbers in the Table are Spearman’s coefficients

There were 36 participants who reported living with caregivers. The caregivers were 2 sisters, 7 children, 4 non-relatives, 17 spouses, 6 spouse and child pairs. Severity of PD, age, and PD duration as well as the scores of SD and dietary modifications are higher in these participants with than in those without support from caregivers, but the difference in BMI is not statistically significant (Table 4). Most, 207/237, participants live with relatives. Only 32 participants reside with children, 112 participants with a spouse only, 48 participants with a spouse and child pair, and 15 with other persons such as sisters. All of the 30 single-living persons are females. The age and severity of PD participants residing with child (ren) only (*n* = 32) are higher than those residing with a spouse and child pair (*n* = 80) as well as with a spouse only (*n* = 112), suggesting that participants rely more on children when the severity of PD increases with age. The logistic analysis showed that use of dietary modifications is related to the presence of caregivers and of children residing with participants. Eating care foods is negatively related to the presence of children residing with participants.

**Table 4:** Characteristics of caregivers at meals and persons residing with patients

	Presence of caregivers at meals			Persons residing with participants (patients)			
	Yes *n* = 36	No *n* = 201	*p* value	Child only *n* = 32	Presence of child *n* = 80	Spouse only *n* = 112	*p* value
Age (years)	73.9 ± 6.8	70.9 ± 8.5	0.0217*	77.7 ± 6.2 a, b	71.0 . ± 8.6 a	70.8 ± 7.0 b	a: *p* = 0.0004*; b: *p* < 0.0001*
PD duration (years)	12.1 ± 7.8	7.6 ± 6.1	0.0022*	10.7 ± 9.3	7.8 ± 5.4	8.0 ± 5.8	ns
Score of SD	12.4 ± 9.5	6.9 ± 7.8	0.0019*	9.2 ± 8.8	7.6 ± 7.8	8.7 ± 9.1	ns
Modification score	1.7 ± 0.9	1.2 ± 0.6	0.0028*	1.5 ± 0.9	1.2 ± 0.6	1.3 ± 0.7	ns
Modified H&Y scale	6.4 ± 1.2	4.3 ± 1.4	<0.0001*	5.1 ± 1.3 c, d	4.4 ± 1.4 c	4.5 ± 1.4 d	c: *p* = 0.030*; d: *p* = 0.039*
BMI	21.3 ± 3.6	22.5 ± 3.6	ns	21.9 ± 4.1	22.3 ± 3.7	22.5 ± 3.5	ns
Goodness of fit for	Presence of caregivers at meals *n* = 36			Child only *n* = 32	Presence of child *n* = 80	Spouse only *n* = 112	
Use of dietary modifications	OR: 6.8, CI: 3.1-15.2, *p* < 0.0001*			ns	OR: 3.4, CI: 1.2-11.4, *p* = 0.024*	ns	
Use of care foods	ns			ns	OR: 0.2, CI: 0.03-0.82, *p* = 0.025*	ns	

“Presence of child” means the number of patients residing with child(ren) plus that residing with child(ren) and spouse. *: significant, *ns*: non-significant

*a*, *b*, *c* and *d* show values where statistical significance as per the *p* values in the very right hand column is reached

Table 5 shows the characteristics of participants based on the types of foods. Of the 11 eating care foods, 10 are female and 6 out of these 11 participants reported eating porridge rice as well. Of the 10 females, 3 reside with child (ren) only, 3 participants with a spouse only and a participant with child (ren) and spouse, 3 participants here are living alone. The gender preponderance for eating care foods is not statistically significant, however ages in participants using care foods are significantly higher (*p* = 0.02) than those of participants eating ordinary rice. When all items other than BMI are considered, participants eating porridge rice only have higher values than those eating ordinary rice only. There are no differences in any of the items here between participants eating care foods with porridge rice or with ordinary rice. The score of SD in participants eating porridge rice is significantly higher than for those eating ordinary rice.

**Table 5:** Characteristics of participant responses based on types of foods

Foods	Ordinary rice &/or only			Porridge rice &/or only		
	Care foods (*n* = 5)	Only (*n* = 172)	Processed side dishes (*n* = 30)	Care foods (*n* = 6)	Only (*n* = 24)	
Items	mean ± S.D.	mean ± S.D.	mean ± S.D.	mean ± S.D.	mean ± S.D.	*p* value
Age	78.3 ± 4.2 a	70.0 ± 8.3 a, b, c	72.8 ± 8.3	77.4 ± 3.6 b	76.3 ± 6.5 c	a: 0.018, b: 0.017, c: 0.0004
PD duration	5.8 ± 4.4	7.6 ± 5.8 a, b	9.9 ± 7.0 a	9.2 ± 6.3	12.3 ± 9.7 b	a: 0.049, b: 0.013
Score of SD	7.1 ± 3.8 a	5.9 ± 6.9 b, c, d	8.9 ± 8.0 b, e	18.2 ± 12.8 c	16.8 ± 9.8 a, d, e	a: 0.04. b: 0.013, c: 0.015, d: <0.0001, e: 0.003,
BMI	23.4 ± 3.4 a	22.7 ± 3.7 b	22.0 ± 3.3	21.2 ± 3.2	20.4 ± 3.2 a, b	a: 0.035, b: 0.003
Modified H&Y scale	4.2 ± 1.3	4.1 ± 1.4 a, b, c	5.0 ± 1.3 a	5.8 ± 0.8 b	5.3 ± 1.3 c	a: 0.001, b: 0.002, c: 0.0005

S.D.: Standard Deviation

*a*, *b*, *c*, *d* and *e* show values where statistical significance as per the *p* values in the very right hand column is reached

For the BMI values, there are no differences between participants eating ordinary rice plus processed side dishes and participants eating porridge rice although the score of SD in participants eating ordinary rice plus processed side dishes is better than in those eating porridge rice. However, BMI values in participants eating porridge rice only are significantly lower than that in participants eating ordinary rice only or ordinary rice plus care foods. No lower BMI was found in participants eating porridge plus care foods compared with participants eating ordinary foods although the score of SD in participants eating porridge rice plus care foods is significantly worse than in those eating ordinary rice only. A higher BMI was found in participants eating ordinary rice plus care foods compared with participants eating ordinary rice only, regardless of the worse SD score, although the differences in BMI and the SD score are not significant. To determine what factors would affect the BMI, a goodness of fit analysis was performed among items such as severity of PD, scores of SD, eating care foods and porridge rice, gender, and age based on Table 5 and among these BMI is significantly related to eating porridge rice only (*p* = 0.003), the data are not shown here.

**Fig. 1:**
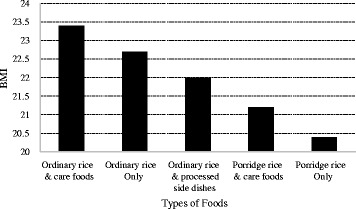
Mean BMI values based on types of foods. Shows mean BMI values as detailed in Table 5

Figure 1 shows relations between BMI as detailed in Table 5 and the types of foods. The BMI becomes lower when changing from ordinary rice to porridge rice. It appears that care foods boost BMI to higher values, like those obtained from ordinary rice only or porridge rice only.

## Discussion

A notable finding of the study here is that dietary modification is practiced irrespective of income level. This finding suggests that outlays for dietary modification are recognized as a very important and essential expense. It is also the least expensive item (compared with care foods and adaptive utensils) of those investigated here.　There is also a statistically significantly lower BMI in participants eating porridge consistency rice, and eating porridge rice is significantly related to the severity of SD and PD. Functional disorders in PD participants would make rehabilitation of swallowing less effective than in participants with other etiologic diseases because disturbances in the oropharyngeal and esophageal phase in swallowing occurs in the early stage of PD [4]. Effects of L-dopa treatment on improvements in the functions of eating and swallowing have not been determined although functioning in the oral phase could improve and result in a better performance of the swallowing function [21]. Therefore, dietary modifications should be recommended in PD patients. The outlays for the dietary modifications are the smallest but the number of participants reporting dietary modifications is the largest among the directly SD-related items, showing that dietary modification plays a very important role to cope with SD (Table 2). Here, soft rice porridge is usually eaten with softened side dishes, suggesting the potential for malnutrition. Participants eating the modified foods would present the possibility of lower nutrition intakes [22]. In the study here the BMI is negatively related to the score of dietary modifications but not to the SD scores (Table 3), suggesting that a texture-modified diet may result in malnutrition expressed as a lower BMI. The BMI here was not significantly correlated with the severity of SD, age, or duration of PD like in other reports [23]. It has also been speculated that reduced energy intake and/or increased energy expended may be associated with body weight loss and lower BMI in PD [24].

The lower BMI here is associated with a lower energy intake caused by eating the porridge rice. In older adults consumption of only few varieties of energy-dense food should be avoided to prevent low BMI even in apparently healthy elderly persons [25], and malnutrition can be reversed with simple dietary education of older adults. While PD is a neurodegenerative disease and it is difficult to compare results based on other causes of SD with the results here, the findings in [24] may be relevant also in this case.

The caregiver is important in preventing lower BMI. The nutritional status of persons assisted by a Home Care Program and 100 % dependent on caregivers is determined by the caregiver educational level, not income [26]. Our report shows that in addition to BMI, incomes are not related to use and costs of SD-related items, however that dietary modification is the most effective activity to cope with SD and that it is related to a lower BMI. Therefore, improvement in the nutritional status in the PD participants (as well as in PD patients in general) could be attained by caregivers and relatives residing with the participants and being provided with nutritional information by dietitians and/or specially trained nurses.

Commercially available care foods are another choice playing a role in preventing malnutrition, and this has been shown for a variety of types of care food products [18]. The logistic analysis showed that BMI is strongly related to types of foods in the Results section above. The SD score in participants eating porridge rice plus care foods suffer from more advanced SD than the participants eating ordinary rice only but differences in BMI among the two groups are not statistically significant (Table 5). Figure 1 shows that a decrease in BMI is lessened by eating care foods in participants with severe SD, suggesting that care foods is of some help in guarding against malnutrition in the older and more severe SD participants. A variety of care foods could serve to provide increased dietary variety to maintain the BMI of those eating ordinary rice or porridge rice [25].

Older respondents and especially females are more likely to make use of care foods (10 females of 11 users). Physical disability may be a source of difficulty in shopping, cooking [27], and changing eating habits among older women [28]. However, here there is no tendency towards lower BMI and body weight loss related to gender. Care foods were shown to prevent malnutrition in the 10 females here in spite of the presence of advanced SD and PD. The number of participants reporting care foods (*n* = 11) is very small compared with that of all of the participants on a texture-modified diet (*n* = 58) likely because of its higher price [18]. More money was spent on care foods than on dietary modifications and the monthly outlays for care foods are the highest among the SD-related costs as shown in Table 2. Nobody reported eating care foods among the 32 participants who were still working as shown in Table 1, and the outlays for care foods are not associated with incomes as shown in Table 3. These results suggest that the need to cope with SD overcomes the higher costs for care foods in the 11 participants (Table 2).

There are two kinds of food supplements: one for additional energy and nutrition and the other for general well-being like with herbal supplements. The former has been shown to be effective but the usefulness of the latter is subject to controversy. The outlays for supplements are related to the score of SD, indicating that participants with more severe SD preferred the supplements (10.2 ± 9.5 vs. 6.9 ± 7.8, *p* < 0.025). The outlays for alternative therapies (like massage or acupuncture) are related to the outlays for supplements, suggesting that participants who use supplements spend more money on alternative*s*. Outlays for alternatives are positively related to the outlays for eating utensils. For participants using adaptive utensils, the SD score was higher than that for participants not using adaptive utensils (10.2 ± 9.6 vs 7.1 ± 7.9, *p* < 0.029). Motor disability makes it difficult to ingest foods, and adaptive utensils may be thought helpful to promote and extend the range of independent eating. Japanese massage has been reported to improve the shoulder range of movement, suggesting relief of muscle stiffness as something that would make eating easier [29].

Table 3 shows that the outlays for dietary modifications are not proportional to other variables as it has the lowest mean value in Table 2. However, the use of dietary modification is important in the management of SD because only the BMI is negatively related to the dietary modification score. The dietary modifications are provided by other persons rather than a result of buying more expensive commercially available foods. Table 4 shows that dietary modifications may be the work of caregivers and/or children rather than by the survey participants and so the cost of dietary modification was not considered critical in the effort to cope with SD. In Japan the spouse and/or daughters are assumed to play a role in caregiving [30]. Due to the costs of increasing care for participants (elderly SD sufferers), members of the family must take responsibility in caring for these participants even when the severity of PD is slight. When the severity of PD increases, the ages in both PD participants and the spouse are also higher (Table 1, Table 4), with the result that spouses find it difficult to care for PD participants in their care, and may finally have to live with child (ren) even when the spouse is alive (Table 4). Table 4 also shows that the PD participants here lived with child (ren) after losing the spouse when participants became older and the severity of PD advanced. As the severity of PD increased, the outlays for the health-care also increase [31]. The micro-costing study here also showed that outlays for coping with SD increase with the severity of PD. Use of care foods which are nourishing but expensive is not related to caregivers but negatively related to the presence of children residing with the participants concerned (Table 4). These findings suggest that participants living with children and/or spouses preferred modified diets, which were provided for them, in preference to care foods, resulting in higher risks of malnutrition.

Although care foods may be used for side dishes due to the higher prices, care foods play an important role in preventing malnutrition as shown in Table 5 and Fig. 1. A Government report on care foods has suggested that one problem is to improve understanding of the utility of care foods [18]. Among the participants interviewed here, there is a considerable number who have only little information on the usefulness of care foods although the number was not tabulated. As mentioned above, education and advice about the usefulness of care foods to caregivers should be provided by dietitians and /or specially trained nurses and everybody expressed hopes that prices of care foods would be cheaper, at a level where more of the survey participants could make use of them to assist in coping with malnutrition.

There are several limitations to the current study. It was shown that care foods may be effective to prevent malnutrition in patients who eat rice porridge. However, only an intervention study could prove the effectiveness of care foods for prevention of malnutrition. It would be desirable for this to be proved by an intervention examination before firm conclusions can be made. Further, in the current study SD-related costs were classified into 5 categories, but the median levels of the SD-related cost were the minimum values of the category. Therefore, it will need further study to distinguish smaller amounts in more detail. Finally, continued research with the participants studied here would be able to show how the results obtained here could affect the various factors considered in this paper.

## Conclusions

To our knowledge, this is the first report with an analysis of resources dedicated to coping with SD in PD participants by qualitative methods. This micro-costing study could suggest behavior patterns that make it possible to cope with decreased QOL and swallowing difficulties. For caregivers at meals, a child was assumed to be the most important person to provide care for PD participants as they become older and as more advanced stages of SD (PD) developed. Porridge rice (rice porridge) which is a kind of modified food was preferable in older participants with advanced stage PD, to cope with the SD. For such participants, the BMI, however, is lower with the porridge rice than with participants relying on ordinary cooked rice. Care foods could be a better choice for warding off malnutrition, however, the number of participants here is very small because of the higher price. Older respondents and especially females (10 females of 11 users) are more likely to make use of care foods. It is a noteworthy finding here that dietary modification occurs regardless of income levels. This finding suggests that dietary modification is recognized as a very important and essential expense. It is also the least expensive item (compared with care foods and adaptive utensils). The presence of caregivers and persons residing with the participants is related to dietary modifications but not related to care foods. Therefore, the availability of dietitians and /or specially trained nurses informing of eating and swallowing would be of benefit for caregivers to provide information of nutrition and advice of nutrition therapies and care foods to cope with SD.

## Abbreviations

BMI, body mass index; DRM, disease-related malnutrition; PD, parkinson’s disease; PG-SGA, patient generated subjective global assessment; QOL, quality of life; SD, swallowing difficulties; SDQ, swallowing difficulty questionnaire

## Additional files

Additional file 1: APPENDIX of Questions Related to SD Costs in the Study. (DOCX 34 kb) Additional file 2: APPENDIX of Questions Related to Personal Matters Here. (DOCX 36 kb) Additional file 3: APPENDIX of Questions Extracted from the SDQ for the Study Here. Fourteen questions (1 to 14) were rated by a four-point (0 to 3) scale (0 for no disability and 3 for severe disability) and one (question 15) was a "yes/no" question (yes was scored 2.5 and no was scored 0.5). (DOCX 35 kb)
