# Supplementary material for: Analysis of resources assisting in coping with swallowing difficulties for patients with Parkinson’s disease: a cross-sectional study
Source: BMC Health Serv Res. 2016 Jul 18;16:276. doi: 10.1186/s12913-016-1467-6 (PMC4949767; doi:10.1186/s12913-016-1467-6)
Supplement: Additional file 1: — APPENDIX of Questions Related to SD Costs in the Study. (DOCX 34 kb) [file 12913_2016_1467_MOESM1_ESM.docx]

APPENDIX of Questions Related to SD Costs in the Study Here

| 1. Do you use commercially available care foods? Yes No  If yes, how much do you spend on care foods per month?  Less than 5 thousand yen (about 40-50 US dollars at 2015 exchange rates), 5 to 10 thousand yen, 10 to 15 thousand yen 15 to 20 thousand yen, or more than 20 thousand yen. |
| --- |
| 2. Do you use dietary supplements such as ginkgo, collagen, or others? Yes No  If yes, what are your monthly outlays for these?  Less than 5 thousand yen (about 40-50 US dollars at 2015 exchange rates), 5 to 10 thousand yen, 10 to 15 thousand yen, 15 to 20 thousand yen, or more than 20 thousand yen. |
| 3. Do you use eating utensils? Yes No  If yes, how much do you spend on eating utensils per month?  Less than 5 thousand yen (about 40-50 US dollars at 2015 exchange rates), 5 to 10 thousand yen, 10 to 15 thousand yen, 15 to 20 thousand yen, or more than 20 thousand yen. |
| 4. Which do you commonly eat: ordinary cooked rice or porridge rice?  Which do you commonly eat cut-up, softened, paste foods or other foods such as processed side dishes?  What are your monthly outlays for dietary modifications of food?  Less than 5 thousand yen (about 40-50 US dollars at 2015 exchange rates), 5 to 10 thousand yen, 10 to 15 thousand yen, 15 to 20 thousand yen, or more than 20 thousand yen. |
| 5. Do you use alternative therapies such as acupuncture, massage or others?  Yes No  If you use it, how much do you spend per month?  Less than 5 thousand yen (about 40-50 US dollars at 2015 exchange rates), 5 to 10 thousand yen, 10 to 15 thousand yen, 15 to 20 thousand yen, or more than 20 thousand yen. |
