# Supplementary material for: Analysis of resources assisting in coping with swallowing difficulties for patients with Parkinson’s disease: a cross-sectional study
Source: BMC Health Serv Res. 2016 Jul 18;16:276. doi: 10.1186/s12913-016-1467-6 (PMC4949767; doi:10.1186/s12913-016-1467-6)
Supplement: Additional file 2: — APPENDIX of Questions Related to Personal Matters Here. (DOCX 36 kb) [file 12913_2016_1467_MOESM2_ESM.docx]

APPENDIX of Questions Related to Personal Matters Here

| 1. What is your annual income?  Below 2 million yen (sixteen to twenty thousand US dollars), 2 to 4 million yen, 4 to 6 million yen, or above 6 million yen. |
| --- |
| 2. Do you work? |
| 3. For meals do you need caregivers? |
| 4. Who reside with you? Single Brothers/Sisters Spouse Child(ren) |
| 5. What is your gender? Male Female |
| 6. How old are you? |
| 7. What is your height and weight? |
| 8. When did you become aware of PD symptoms? |
| 9. What is your PD score on the Hoehn & Yahr scale? |
