# Supplementary material for: Analysis of resources assisting in coping with swallowing difficulties for patients with Parkinson’s disease: a cross-sectional study
Source: BMC Health Serv Res. 2016 Jul 18;16:276. doi: 10.1186/s12913-016-1467-6 (PMC4949767; doi:10.1186/s12913-016-1467-6)
Supplement: Additional file 3: — APPENDIX of Questions Extracted from the SDQ for the Study Here. Fourteen questions (1 to 14) were rated by a four-point (0 to 3) scale (0 for no disability and 3 for severe disability) and one (question 15) was a "yes/no" question (yes was scored 2.5 and no was scored 0.5). (DOCX 35 kb) [file 12913_2016_1467_MOESM3_ESM.docx]

APPENDIX of Questions Extracted from the SDQ for the Study Here

|  | Questions |  | Questions |
| --- | --- | --- | --- |
| 1 | Do you experience difficulties in chewing solid food, like apples, cookies, or crackers? | 8 | Do you experience difficulty in swallowing pureed food? |
| 2 | Are there any food residues in your mouth, cheeks, under your tongue, or sticking to your palate after swallowing? | 9 | While eating, do you feel as if a lump of food is stuck in your throat? |
| 3 | Does food or liquids come out of your nose when you eat or drink? | 10 | Do you cough while drinking liquids? |
| 4 | Does chewed-up food dibble from your mouth? | 11 | Do you cough while swallowing solid food? |
| 5 | Do you feel you have too much saliva in your mouth; do you drool or have difficulty swallowing your saliva? | 12 | Do you experience changes in your voice, such as hoarseness or reduced intensity immediately after eating or drinking? |
| 6 | Do you need to swallow chewed-up food several times before it goes down your throat? | 13 | Other than during meals, do you experience coughing or difficulty breathing as a result of saliva entering your windpipe? |
| 7 | Do you experience difficulties in swallowing solid food (i.e. do apples or crackers get stuck in your throat?) | 14 | Do you experience difficulty in breathing during meals? |
| 15 | Have you suffered from respiratory infections (pneumonia, bronchitis) during the past year? | | |
